# IL-12 Signaling Contributes to the Reprogramming of Neonatal CD8^+^ T Cells

**DOI:** 10.3389/fimmu.2020.01089

**Published:** 2020-06-05

**Authors:** Darely Y. Gutiérrez-Reyna, Alejandra Cedillo-Baños, Linda A. Kempis-Calanis, Oscar Ramírez-Pliego, Lisa Bargier, Denis Puthier, Jose D. Abad-Flores, Morgane Thomas-Chollier, Denis Thieffry, Alejandra Medina-Rivera, Salvatore Spicuglia, Maria A. Santana

**Affiliations:** ^1^Centro de Investigación en Dinámica Celular (IICBA), Universidad Autónoma del Estado de Morelos, Cuernavaca, Mexico; ^2^Aix-Marseille University, TAGC, INSERM UMR1090, Marseille, France; ^3^Equipe Labélisée Ligue Contre le Cancer, Marseille, France; ^4^Institut de Biologie de l'École Normale Supérieure (IBENS), Département de Biologie, École Normale Supérieure, CNRS, INSERM, Université PSL, Paris, France; ^5^Laboratorio Internacional de Investigación sobre el Genoma Humano, Universidad Nacional Autónoma de Mexico, Juriquilla, Mexico

**Keywords:** neonatal T cells, CD8^+^ T cells, RNA-sequencing, IL-12, T cell activation, neonatal immunity

## Abstract

Neonates are highly susceptible to intracellular pathogens, leading to high morbidity and mortality rates. CD8^+^ T lymphocytes are responsible for the elimination of infected cells. Understanding the response of these cells to normal and high stimulatory conditions is important to propose better treatments and vaccine formulations for neonates. We have previously shown that human neonatal CD8^+^ T cells overexpress innate inflammatory genes and have a low expression of cytotoxic and cell signaling genes. To investigate the activation potential of these cells, we evaluated the transcriptome of human neonatal and adult naïve CD8^+^ T cells after TCR/CD28 signals ± IL-12. We found that in neonatal cells, IL-12 signals contribute to the adult-like expression of genes associated with cell-signaling, T-cell cytokines, metabolism, and cell division. Additionally, IL-12 signals contributed to the downregulation of the neutrophil signature transcription factor CEBPE and other immaturity related genes. To validate the transcriptome results, we evaluated the expression of a series of genes by RT-qPCR and the promoter methylation status on independent samples. We found that in agreement with the transcriptome, IL-12 signals contributed to the chromatin closure of neutrophil-like genes and the opening of cytotoxicity genes, suggesting that IL-12 signals contribute to the epigenetic reprogramming of neonatal lymphocytes. Furthermore, high expression of some inflammatory genes was observed in naïve and stimulated neonatal cells, in agreement with the high inflammatory profile of neonates to infections. Altogether our results point to an important contribution of IL-12 signals to the reprogramming of the neonatal CD8^+^ T cells.

## Introduction

The neonate population is at high risk of infection by intracellular pathogens, which cause severe diseases and a high mortality rate (1). CD8^+^ T cells are responsible for the elimination of dysfunctional, infected, or stressed cells through antigen-dependent mechanisms. Naïve T cell activation takes place in lymph nodes when T cells recognize specific antigens presented by dendritic cells. The sole presence of antigens is not sufficient, however, to induce priming (2). Instead, to induce cytotoxic T lymphocyte-mediated immunity, antigen recognition must occur in the context of co-stimulation and pro-inflammatory cytokines, particularly interleukin (IL)-12 or IFNα/β (3). Although both cytokines induce the activation and differentiation of CD8^+^ T cells into effector cells, IL-12 signals induced the expression of a larger number of genes, a higher level of IFNγ, and was able to restore the functionality of exhausted T cells (3–5).

Neonatal CD8^+^ T cells have unique characteristics that comply with the tolerance requirements of birth transition (6). However, they leave neonates highly susceptible to intracellular pathogen infections. Neonatal T cells have a low production of cytokines and their response is skewed into the Th2 and tolerance phenotypes, although they may produce an adult-like response, with a very high threshold of activation (7–9). We have previously shown that neonatal CD8^+^ T cells have a unique transcriptomic and epigenetic signature, characterized by a lower expression of cytotoxic- and activation-related genes, and a bias toward innate immunity, particularly neutrophil-like inflammation (10). A recent review summarizes the altered characteristics of neonatal CD8^+^ T cells (6).

Production of IL-12 is much lower in neonatal dendritic cells (11) and increases steadily during childhood. At 12 years of age, it is still below the adult levels (12). In addition to antigen recognition and CD28 co-stimulation, recent studies have demonstrated that inflammatory cytokines, particularly IL-12, or IFN α/β, are necessary as a third signal to authorize the differentiation of neonatal CD8^+^ T cells (13). It has also been reported that, in neonatal cells, IL-12, and IFNγ gene promoters are in a silent state, due to hyper methylation (11, 14, 15). Instead of producing IL-12, composed by the p35 and p40 subunits, the neonatal dendritic cells produce the related cytokine IL-23, composed by the p19 and p40 subunits, and the neonatal T cells produce the IL-23 receptor upon stimulation. IL-23 cytokine is an important component of the Th17 profile (15), thereby fostering an inflammatory phenotype.

Although the addition of IL-12 to the stimulatory conditions have been shown to contribute to the functional maturation of cytotoxic T lymphocytes, the underlying molecular mechanisms have not been described yet. In this work, we explored the transcriptomic changes occurring after activation of neonatal CD8^+^ T cells through the TCR in the presence or absence of IL-12. We aimed to evaluate, under high stimulatory conditions, whether the neonatal cells, with a very unique transcriptome, are able to reprogram their gene expression into a profile more similar to that of adult effector cells. We found that in neonatal CD8^+^ T cells, IL-12 is an important signal for the induction of the characteristic cytotoxicity and effector genes expressed in adults. Furthermore, IL-12 signals contribute to the activation of metabolic and cytoskeleton pathways that are permissive for the clonal expansion of the cells, as previously observed (13). A few genes, characteristic of the neutrophil-like inflammation (10), such as the neutrophil signature transcription factor CEBPE and defensin A, were down-regulated by IL-12 signals. Furthermore, these changes were associated with the methylation of the promoters of genes relevant in this signaling cascade. Altogether our results demonstrated that IL-12 contributes to the transcriptional reprogramming of neonatal CD8^+^ T cells, presumably through epigenetic mechanisms.

## Materials and Methods

### Cell Preparation and Stimulation

Leukocyte concentrates were obtained from healthy adult donors from Centro Estatal de la Transfusión Sanguínea in Cuernavaca, Mexico. Cord Blood was collected and processed immediately after birth and before placenta expulsion from vaginal deliveries of full-term healthy newborns, with informed mother's consent. Ethical approval was obtained from Servicios de Salud Morelos (CONBIOÉTICA-17-CEI-001-20160329), Mexico. Peripheral Blood Mononuclear Cells (PBMCs) and Cord Blood Mononuclear Cells (CBMCs) were obtained by density centrifugation with Lymphoprep (Axis-Shield; Dundee, UK). Total CD8^+^ T cells were purified with RosetteSep™ Human CD8^+^ T Cell Enrichment Cocktail (StemCell Technologies; California, USA). After that, we depleted Memory CD8^+^ T cells with Protein A/G magnetic Dynabeads® (10 mg/mL, Thermo Fisher Scientific; Massachusetts, USA), loaded with 1 μg of antibodies against anti-CD45RO (UCH-L1, TONBO biosciences; California, USA), and anti-CD44 (IM7, TONBO biosciences; California, USA). Contaminant neonatal cells were removed with protein A/G pearls, loaded with 0.5 μg anti-CD11b (ICRF44, TONBO biosciences; California, USA). This technique provided us with at least 96% naïve CD8^+^ T cells, verified for each sample by flow cytometry. CD8^+^ T cells were cultured in RPMI 1640 medium (Thermo Fischer Scientific; Massachusetts, USA), supplemented with 2 mM glutamine, commercial antibiotics (50 U/mL penicillin, 50 μg/mL streptomycin), and 5% fetal calf serum. Cells were activated with 1 μg/mL anti-CD3 (OKT3, TONBO biosciences; California, USA) and 1 μg/mL anti-CD28 (CD28.2, TONBO biosciences; California, USA), cross-linked with 1 μg/mL goat anti-mouse IgG (Jackson ImmunoResearch), and incubated for 36 h, under 5% CO2 at 37°C. IL-12 (10 ng/mL) (IL-12; RPX-Pro^TM^ Recombinant, TONBO biosciences; California, USA) was added simultaneously when indicated.

### RNA Preparation and RT-qPCR

Total RNA was extracted using TRIZOL® Reagent (Thermo Fisher Scientific; Massachusetts, USA), following the manufacturer's protocol. The quality was evaluated with a Bioanalyzer nano-RNA Kit (Thermo Fisher Scientific; Massachusetts, USA) for the RNA-seq protocols (RNA integrity value over 8.0) and with standard agarose gels for qPCR analysis. cDNA was synthesized by RNA reverse-transcription using the RevertAid Reverse Transcriptase cDNA Synthesis kit (EP0441, Thermo Fisher Scientific; Massachusetts, USA). The primers were designed using NCBI mRNA reference sequence and Primer-Blast (NCBI), taking care to introduce an exon-exon junction. Real-time qPCR was performed in StepOne thermocycler (Thermo Fischer Scientific; Massachusetts, USA) using Power SYBR Green PCR Master Mix (K0221, Thermo Fisher Scientific; Massachusetts, USA). Oligonucleotide sequences are shown in Supplementary Table 1. β2-microglobulin was used as the reference gene in the mRNA expression. The relative expression was obtained with V2.3 software and gene expression was determined using the standard curve method for each gene.

### Methylated DNA Immunoprecipitation

For Methylated DNA Immunoprecipitation (MeDIP), cells were lysed with Tween 20 (1:200) in the presence of 1 μL proteinase K (20 mg/ml, Zymo Research; California, USA) and incubated at 55°C for 1 h. The proteinase K was heat-inactivated and genomic DNA (gDNA) was precipitated with a mixture of 1 vol Isopropanol and 1:10 (vol/vol) 3 M sodium acetate (pH 5.2). DNA pellets were resuspended and cleaned using the Expin^TM^ PCR SV Kit (GeneAll® Seoul, KR), according to the manufacturer's protocol. gDNA was sonicated to produce random 200–600 bp fragments. Two micrograms from each gDNA sample were diluted with 225 μL of TE Buffer [10 mM Tris-HCL (pH 8), 1 mM EDTA] and incubated at 95°C for 10 min, and then in 25 μL of 10X IP Buffer (100 mM sodium phosphate (pH 7.0); 1.4 M NaCl;0.5% Triton X-100), and 2 μg of anti-5mC antibody (1 μg/μL, Zymo Research; California, USA) at 4°C over-night under rotation. The Immunoprecipitated gDNA was recovered and we added 0.2 mg per sample of prewashed Dynabeads® Protein A/G (10 mg/mL, Thermo Fisher Scientific; Massachusetts, USA) loaded with 2 μg of Rabbit anti-mouse IgG(H+L) (816700, ZyMaxTM Invitrogen; California, USA). The immunoprecipitated sample was recovered, proteins were eliminated with Proteinase K and the DNA was cleaned again with Expin^TM^ PCR SV Kit. The enriched methylated DNA was analyzed by amplifying selected gene promoters with qPCR, in comparison with the same amount of non-precipitated DNA (Input). The promoter region of β-actin was used as negative control and that of EBF1 was used as a positive control, giving the expected results (not shown). To obtain the primers for PCR amplification of promoter regions, we selected regions 1,000 upstream and 200 downstream after TSS that corresponded to CpG Islands, selected from the Eukaryotic Promoter Database EPD (http://epd.vital-it.ch/) and the Methyl Primer Express® Version 1.0 software. Primers were then designed with Primer-Blast, NCBI. All primer sequences are included in Supplementary Table 1. For all PCR amplification products, we used the standard curve method.

### RNA-seq and Library Preparations

Total RNA from CD8^+^ T cells was extracted using TRIZOL® Reagent, following the manufacturer's protocol. The quantity and quality of samples from all conditions were verified on a Fragment analyzer (Agilent; California, USA); all samples had an RNA integrity number ≥ 8.1. RNA-seq libraries were constructed at the Transcriptomic and Genomic Marseille Luminy Platform (Aix-Marseille University, TAGC) using the TruSeq mRNA Library Prep Kit v2 (Illumina; California, USA). Libraries were paired-end sequenced on an Illumina NextSeq 500 sequencer.

### RNA-seq Data Processing, Differential Expression, and Clustering

After sequencing, raw data were obtained in the fastq format. The quality of the data was evaluated with FastQC (http://www.bioinformatics.babraham.ac.uk/projects/fastqc). Adapters and low-quality sequences were trimmed or discarded using Trimmomatic (16). Quality check using FastQC was performed again on the trimmed sequences. Then reads were aligned to the human reference genome GRCh38/hg38 to generate the Binary Alignment Map (BAM) files with STAR (v2.4.2a) (17). Briefly, transcripts discovery was performed using Cufflinks (v2.2.1) with the “library-type” argument set to fr-firstrand, and a GTF file obtained from GENCODE (“Comprehensive gene annotation,” vM1) provided as the genomic annotation. GTF files were produced by Cufflinks and combined using Cuffmerge. Only *de novo* transcripts with counts >0 in at least one RNA-seq sample were kept for subsequent analyses. These *de novo* transcripts were combined with the GENCODE GTF file to produce the final genomic annotation used with FeatureCounts (v1.4.6-p4) for quantification (18). The R package, DESeq2 (v1.6.3) was used to screen differentially expressed genes and normalization of the count data (19). Differences were considered statically significant if adjusted *p*-value was <0.05 and log_2_ fold change ≥ 2 was further considered, unless otherwise specified. The differentially expressed genes on basal conditions were subjected to *k*-means clustering using the Euclidean distance method. The expression of genes for each condition within the same cluster similarly responded to stimulation. *K*-means = 12 was selected because this number represented fairly the different expression profiles at the base level and in response to stimulation. This adjustment was performed manually, comparing 8, 10, 12, 14, and 20 *k*-means clusters for patterns of expression. We chose 12 because these clusters better grouped the different patterns of gene expression, while limiting the presence of similar clusters with different intensities. Raw and processed RNA-seq data were deposited on the Gene Expression Omnibus (GEO; GSE144108). Gene expression was visualized using the IGV genome browser.

### Functional Enrichment Analysis and Pathways

The differentially expressed genes for each condition were loaded in the WEB-based Gene Set Analysis Toolkit (WebGestalt) (20) to identify enriched pathways. The top 20 Reactome pathways having adjusted *p* < 0.05 were selected. Reactome pathways, Kyoto Encyclopedia of Genes and Genomes (KEGG) pathways and Gene Ontology terms (GO) biological process were obtained from the Database for Annotation, Visualization and Integrated Discovery (DAVID 6.8, https://david.ncifcrf.gov/) software (21).

### Statistical Analysis for RT-qPCR

Results were analyzed with the GraphPad Prism software (GraphPad; California, USA). Statistical significance was evaluated by the two-tailed unpaired Student's *t*-test; differences with *p* < 0.05 were considered significant.

## Results

### IL-12 Signals Contribute to the Transcriptional Reprogramming of Neonatal CD8^+^ T Cells

**Figure 1:**
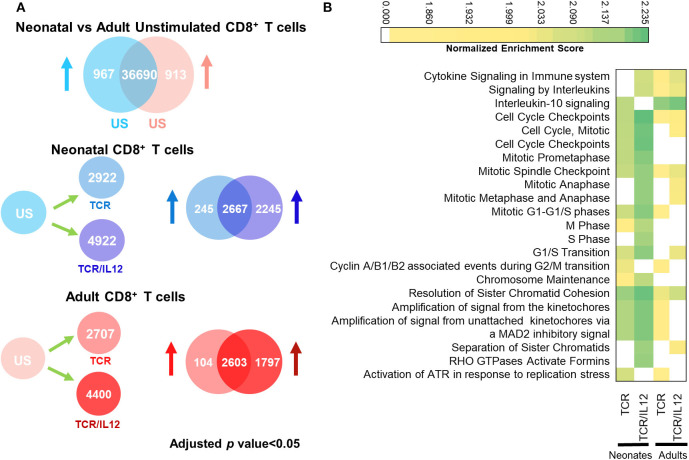
TCR or TCR/IL-12 induced response in neonatal and adult CD8^+^ T cells. **(A)** Number of differentially expressed Genes (adjusted *p* < 0.05) between non-stimulated and TCR or TCR/IL-12 neonatal and adult CD8^+^ T cells. US, unstimulated cells. **(B)** Top 20 significantly enriched REACTOME pathways (adjusted *p* < 0.05) in neonatal and adult naïve CD8^+^ T cells stimulated through the TCR or TCR/IL-12 signals.

To investigate the role of IL-12 on the activation CD8^+^ T cells, we performed RNA-seq analysis of purified naïve CD8^+^ T cells left untreated or activated by cross-linking the CD3 and CD28 molecules (TCR), alone or in the presence of IL-12 (TCR/IL-12) for 36 h. In this first analysis, we included all differentially expressed genes (adjusted *p* < 0.05), as compared to the un stimulated cells (US), without any fold change cutoff, in accordance with the GSEA pathway analysis, which considers all significant changes. Comparison of triplicate samples of naïve neonatal and adult cells displayed 967 and 913 genes significantly overexpressed in neonatal and adult cells, respectively (adjusted *p* < 0.05) (Figure 1A). In agreement with our previous report, in which we showed that neonatal cells had a higher homeostatic proliferation and were biased toward neutrophil-like inflammation (10), we found that pathways in neonatal cells were biased toward cell cycle and innate immunity (Supplementary Figure 1). In contrast, no enriched pathways were obtained from the adult naïve CD8^+^ T cells. After TCR stimulation, 2,922 and 2,707 genes were upregulated (adjusted *p* < 0.05) in neonatal and adult cells, respectively. As expected, TCR stimulated genes in adult cells were associated with immune response, while those of neonates were still biased toward cell cycle and IL-10 signaling (Figure 1B), in agreement with the tolerant phenotype of neonatal cells. Remarkably, in both populations, TCR/IL-12 stimulation induced the significant expression of almost the double of genes, as compared to TCR stimulation (4,922 and 4,400 genes in neonatal and adult cells, respectively). Moreover, the pathways induced by TCR/IL-12 included cytokine signaling pathways. This is indicative of the maturation and activation of the neonatal cells, similar to the adult ones. Remarkably, only in the neonatal cells, IL-10 pathway was downregulated in response to TCR/IL-12 stimulation. This was surprising, as IL-10 itself was induced with this stimulus (**Figure 3B**). When observing the heatmap of the IL-10 pathway (Supplementary Figure 2), it was noteworthy that only in the neonatal cells, TCR/IL-12 signals inhibited the expression of the α and β chains of the IL-10 receptor (IL10RA and IL10RB, respectively) and the signaling kinase JAK1, presumably lowering the high threshold for neonatal T cell activation (8). Thus, IL-12 co-stimulation has a strong synergistic effect on TCR signals in neonatal and adult cells, suggesting a contribution of IL-12 signals toward neonatal cells maturation and to the full activation in both cell populations.

**Figure 2:**
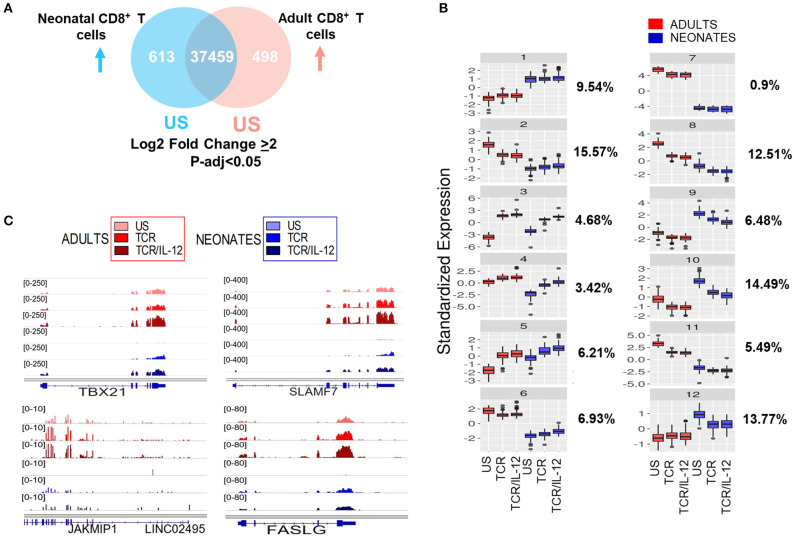
Basal levels and response patterns of differentially expressed genes between neonatal and adult CD8^+^ T cells. **(A)** Venn diagram of differentially expressed genes between naïve neonatal and adult naïve CD8^+^ T (adjusted *p* < 0.05 and Log_2_ fold change ≥ 2). **(B)** *k*-means clustering of the pattern expression of genes, based in their base levels and response to TCR or TCR/IL-12 treatment in neonatal and adult CD8^+^ T cells. The percentage of genes belonging to each cluster is shown to the left. **(C)** Genome browser screenshots of RNA-seq data, showing four genes (*TBX21, SLAMF7, JAKMIP1*, and *FASLG*) from Cluster 4 that reached the adult's expression level in neonates stimulated cells.

Next, we analyzed the patterns of gene expression changes after TCR or TCR-IL12 stimulation, considering only highly significant differential genes (adjusted *p* < 0.05 and log_2_ fold change ≥ 2) after TCR or TCR-IL12 stimulation (Figure 2A). To identify the main gene expression changes after TCR or TCR/IL-12 stimulation, we performed *k*-means analysis, to classify the response pattern of the differentially expressed genes between naïve CD8^+^ T cells from neonate and adult donors into 12 clusters (Figure 2B). Clusters 1 and 7 correspond to genes that remained largely unchanged (10.44%). Clusters 2–6 represent genes that were induced in neonatal cells by TCR, but which expression was increased by the addition of IL-12 (37%). Clusters 8–12 represent genes down-regulated by stimulation of the neonatal cells (53%). We observed that IL-12 co-stimulation leads to an improved response of the neonatal cells, leading to an increased similarity with those of adults. On the one hand, cluster 3 represents a set of genes that upon IL-12 co-stimulation reached the level of those of stimulated adult cells. This cluster includes genes related to cytotoxicity function and will be further discussed in the next section. Cluster 4 represents a set of neonatal-expressed genes reaching adult basal levels upon TCR/IL-12 stimulation. These include important functional genes such as *TBX21* (T-bet), *SLAMF7, FASL*, and *JAKMIP1* (Figure 2C). On the other hand, genes in clusters 9 and 10 diminished their expression to adult's levels and correspond to immaturity-related genes. Cluster 5, representing 6.21% of the genes and enriched in inflammatory genes, displayed an exacerbated response to stimulation in neonatal cells. It is thus clear that IL-12 co-stimulation not only induces, but also represses gene expression, suggesting a transcriptional reprogramming of the neonatal cells.

### IL-12 Signals Induced the Expression of Cytotoxicity and Cell Signaling Genes

**Figure 3:**
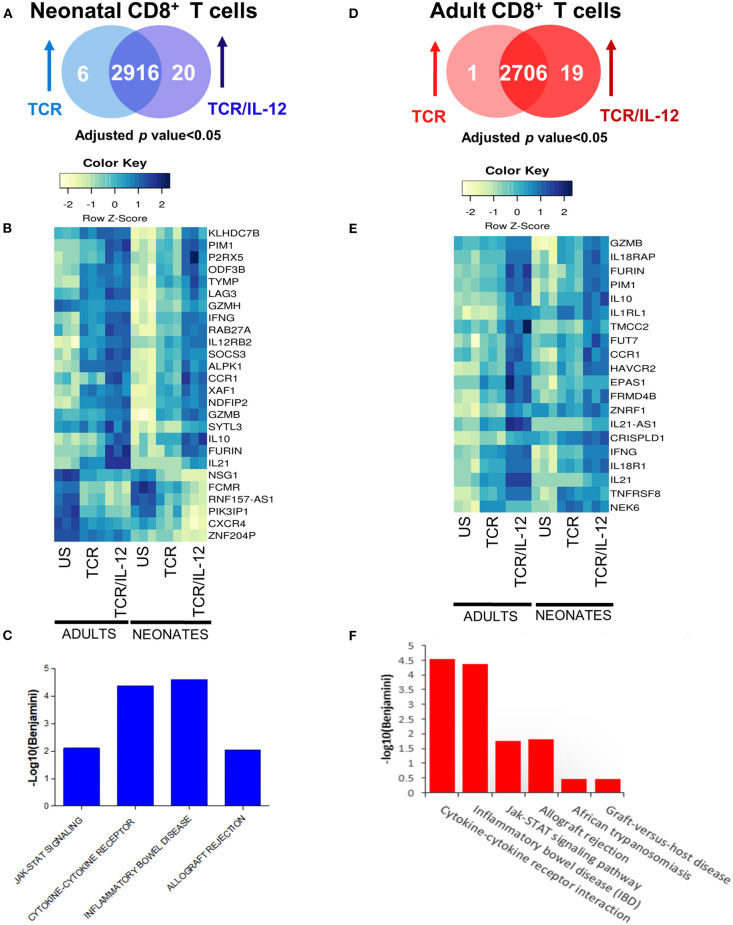
Comparison between TCR and TCR/IL-12 response genes of neonatal and adult naïve CD8^+^ T cells. **(A,D)** Venn diagrams **(B,E)** heatmaps of induced genes after TCR or TCR/IL-12 treatment of neonatal (left) and adult (right) CD8^+^ T cells (adjusted *p* < 0.05). **(C,F)** Enriched KEGG pathways returned by the DAVID software for the differentially expressed genes in neonatal (left) and adult (right) CD8^+^ T cells.

To better understand the specific role of IL-12 signals in neonatal cells, we identified the genes that were significantly induced by TCR/IL-12 stimulation as compared with those only stimulated by TCR signals (Figure 3). We therefore asked which genes were significantly regulated (adjusted *p* < 0.05) in neonatal (Figure 3A) and adult cells (Figure 3D) by the TCR/IL-12 stimulation as compared to the TCR-induced genes. In the neonatal cells, 20 genes were induced by TCR/IL-12 signals as compared to those induced by the TCR alone (Figure 3B). These induced genes were associated with four KEGG pathways related to inflammation, cytokine function and signaling (Figure 3C). Likewise, 19 genes were induced by TCR/IL-12 as compared to TCR conditions in adult cells (Figure 3E), leading to 6 pathways related to inflammation and cytokine signals (Figure 3F). In both cell populations, the IL-12 signals increased the expression of key genes associated with cytotoxicity, *GZMB*, and *GZMH*; inflammation, *IL21;* control of excessive inflammation, *IL10*; activation related genes, S*OCS3* and *LAG3*; sensing IL-12 signals, *IL-12RB;* and the hallmark cytokine for CD8^+^ T cell activation, *IFNG*. IFNγ is epigenetically controlled by a long non-coding RNA named *NeST* or *IFNG-AS1* (22, 23), located upstream of *IFNG* locus. Supplementary Figure 3 shows that, unlike naïve adult samples, the *IFNG-AS1* is highly expressed in the non-stimulated neonatal cells and is down-regulated by stimulation, particularly in the presence of IL-12, in agreement with an epigenetic signature of neonatal CD8^+^ T cells that is modified by TCR/IL-12 signals. The chemokine receptor CCR1 was also induced. The expression of *CCR1* is increased by IFNγ and allows the migration of the cells into inflamed tissues. Additionally, CCR1 promotes a flipping of the Th1/Th2 balance toward the Th1 phenotype (24). Six genes were downregulated by IL-12 addition in the neonatal cells, including that coding for the PI3K inhibitor PIK3IP1, allowing the activation of the PI3K pathway and thus glycolysis, further described in the next section. The chemokine receptor CXCR4 was also down-regulated by stimulation in the presence of IL-12. CXCR4 is related to bone marrow homing (25). In Supplementary Figure 4, we present the RNA-seq profiles of a selection of genes that were found overexpressed by the addition of IL-12 to the TCR stimulation. To ascertain the validity of this finding, we performed RT-qPCR analysis of these genes using independent samples (Supplementary Figure 4B). In all cases, the pattern of expression observed by the RT-qPCR analysis replicated the pattern of expression observed by the RNA-seq data (Supplementary Figure 4A). Altogether, these data explain the functional improvement described for neonatal CD8^+^ T cells, upon IL-12 addition (13).

### IL-12 Signals Induced the Expression of Metabolism and Cell Function Genes

**Figure 4:**
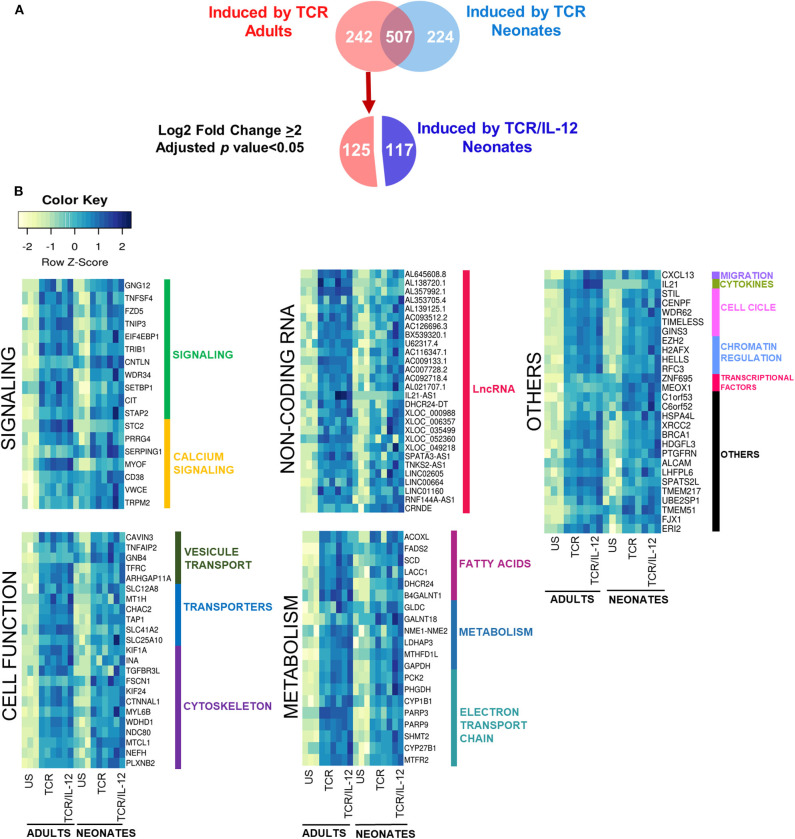
Genes induced by TCR signals in adult naïve CD8^+^ T cells, but only induced by TCR/IL-12 in the neonatal cells. **(A)** Venn Diagram showing the genes induced by TCR in adult cells that are only induced by TRC/IL12 in the neonatal cells, and **(B)** heatmaps of genes induced by TCR in adults CD8^+^ T cells, that only were induced by TCR/IL-12 in neonatal CD8^+^ T cells (adjusted *p* < 0.05 and log_2_ fold change ≥ 2), bars on the right display manual annotations of the functional categories.

To further explore the role of IL-12 in the functionality of neonatal cells, we evaluated the genes whose expression was significantly increased by TCR in adult cells, but in neonates only reached high statistical significance by TCR/IL-12 stimulation (Figure 4). That is, we selected the 242 genes that were strongly induced by TCR stimulation only in adult cells (adjusted *p* < 0.05, log_2_ fold change ≥ 2), and asked which ones where significantly induced by the TCR/IL-12 signals, but not by TCR signal compared to the unstimulated conditions with a log_2_ fold change ≥ 2. A total of 117 were recovered in the neonatal cells by the addition of IL-12 to the stimulatory conditions (Figure 4A). These genes are predominantly involved in cell signaling, cytoskeleton, and cell transport and metabolism (Figure 4B). T cell activation is associated with a metabolic increase, particularly in glycolysis. As expected, glycolysis-related genes were upregulated by TCR/IL-12 stimulation, along with genes involved in fatty acid synthesis, transport chains, ATP synthesis, glucose metabolism, and other transporters. Several non-coding RNAs, mostly associated with genes involved in RNA maturation, were also induced in the presence of IL-12 (Supplementary Table 2). Among the LncRNAs induced, we found *AL353705.4*, related to the splicing factor SCAF1; *AL138720.1*, associated with the polyadenylate binding protein PABPN1; and *AL645608.8* and *AL139125.1*, both associated with the Uridylyl Transferase 7, TUT7. Cell cycle and cell division related genes were already induced by TCR signals in the neonatal cells (Supplementary Figures 5A,B). They were associated with the biological process related to Mitosis (Supplementary Figure 5C) and validated by RT-qPCR in independent samples (Supplementary Figure 5D). However, the increased expression in metabolism-related genes, together with an increase in genes related to membrane growth and fusion and cytoskeleton proteins, specifically found in the TCR/IL-12 co-stimulation conditions, could explain the high clonal expansion of the neonatal cells, observed only in the presence of IL-12 (13).

### IL-12 Signals Repress Immaturity Related Genes of the Neonatal CD8^+^ T Cells

**Figure 5:**
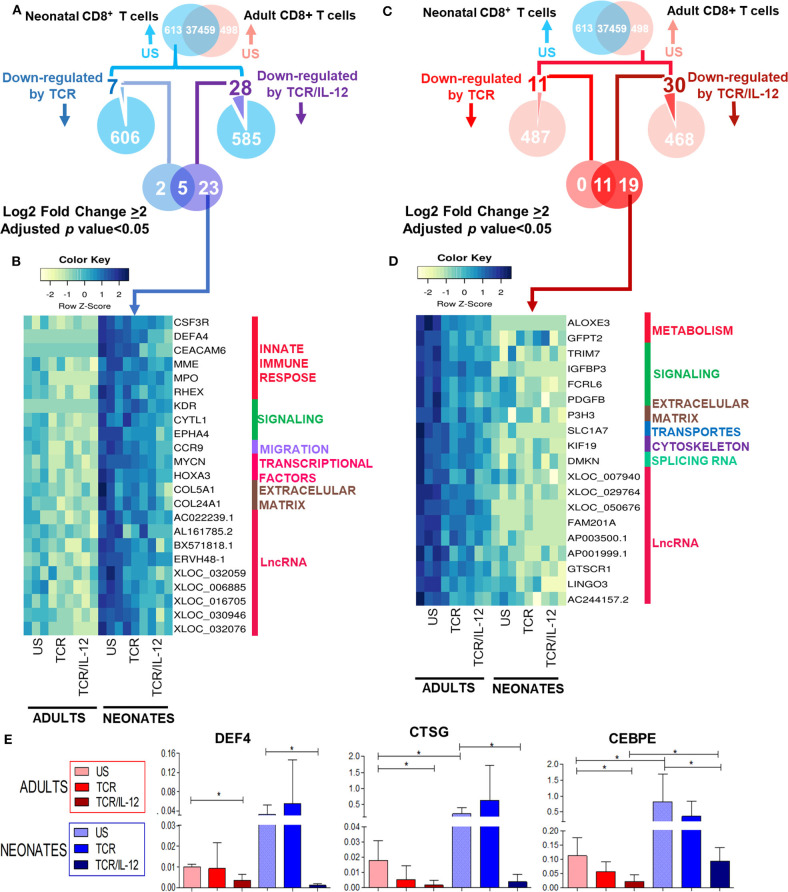
Genes significantly downregulated by TCR/IL-12 signals in neonatal and adult naïve CD8^+^ T cells. **(A,C)** Venn Diagrams showing the genes overexpressed genes in the neonatal **(A)** and adult **(C)** cells but down-regulated by TCR/IL-12. **(B,D)** heatmaps of genes significantly downregulated by TCR/IL-12 in neonatal **(B)** and adult **(D)** CD8^+^ T cells (adjusted *p* < 0.05 and log_2_ fold change ≥ 2), bars on the right display manual annotations of functional categories. **(E)** RT-PCR evaluations of selected significantly down-regulated genes in independent samples (*n* = 5) of neonatal cells, after TCR or TCR/IL-12 treatment, normalized to β2-microglobulin. Data presented are means ± standard deviations. Statistical significance was assessed by a Student's *t*-test (unpaired; *p < 0.05).

Neonatal CD8^+^ T cells overexpress genes related to neutrophil-like inflammation, innate immunity, and T cell precursors, as we show in Supplementary Figure 1 and in our previous report (10). Therefore, we asked whether IL-12 co-stimulation could repress this immature transcriptional signature, as suggested by our *k*-means analysis (Figure 2, clusters 9, 10, and 12). To address this issue, we selected the genes that were highly overexpressed in the neonatal cells after TCR or TCR/IL-12 stimulation, as compared to the unstimulated cells (adjusted *p* < 0.05 and log_2_ fold change > 2) (Figure 5A). We identified a set of 23 genes, overexpressed in naïve neonatal CD8^+^ T cells that were specifically repressed by TCR/IL-12 stimulation (Figure 5B). These genes were associated with the innate immune response (Figure 5C), such as the metalloprotease gene *MPO* and *DEFA4*, associated with antimicrobial activity and migration into intestinal mucosa (CCR9) (26). The *HOXA3* transcription factor, which is expressed in early hematopoietic progenitors and subsequently down-regulated during early T cell differentiation (27–29), was also found highly expressed in the neonatal cells. Adding IL-12 signals to the stimulatory conditions resulted in the downregulation of this gene. Neonatal cells overexpress major neutrophil response genes, such as *CEBPE* and Cathepsin G (*CTSG*). These genes were not found significantly downregulated in this cluster, because the analysis was very stringent. However, using RT-qPCR on independent samples, we show that these genes were also downregulated by TCR/IL-12 signals (Figure 5E). For comparison, we performed the same analysis for the overexpressed genes in the naïve adult cells over those of the neonatal cells (Figure 5D). The genes downregulated by IL-12 were involved in lipid and glutamine metabolism (*ALOXE3, GFPT2*), ubiquitin signaling (*TRIM7*), and other cell functions. In Supplementary Figure 6, we present the same analysis, applying a log_2_ fold change cutoff of 1, to increase the sensitivity of the analysis. We obtained 44 and 53 genes specifically repressed by the TCR/IL-12 conditions in the neonatal and adult cells, respectively. The same pattern of functions was obtained.

Altogether, the addition of IL-12 signals results not only in the induction of characteristic genes of the CD8^+^ T cell response but also in the downregulation of immaturity-related genes in the neonatal cells.

### The Inflammatory Profile of Neonatal CD8^+^ T Cells Is Not Fully Repressed by IL-12 Co-stimulation

We next analyzed the genes from *k*-means cluster 1 that were overexpressed in the un-stimulated neonatal cells as compared to adult cells and did not change significantly after TCR nor TCR/IL-12 stimulation (Supplementary Figure 7). These genes were associated with transcription factors important for precursor cells, monomeric, and trimeric G proteins, cytoskeleton, cell signaling, innate response, and inflammation. Inflammatory genes included those coding for IL-1 receptor (*IL1R*) and Prostaglandin G/H synthase 1 (*PTGS1*), a key enzyme in prostaglandin synthesis.

**Figure 6:**
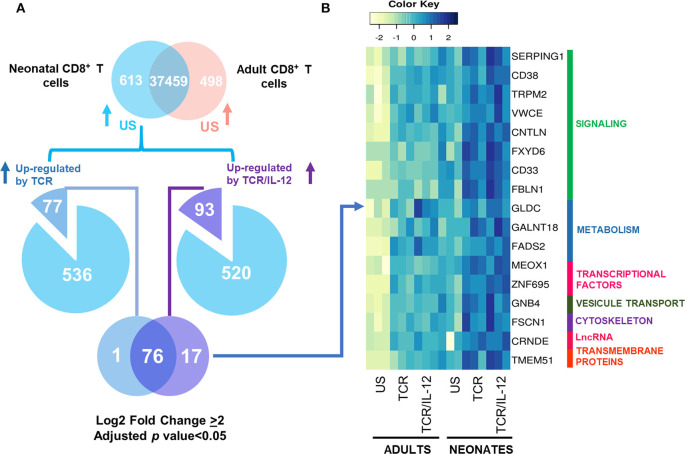
Genes Overexpressed in neonatal CD8^+^ T cells and showing an exacerbated response to TCR/IL-12 signals. **(A)** Venn Diagrams showing the genes analyzed and **(B)** heatmaps with manual annotation of the genes with an exacerbated response to TCR and TCR/IL-12 signals in the neonatal CD8^+^ T cells (adjusted *p* < 0.05 and log_2_ fold change ≥ 2).

Next, we analyzed genes whose expression was already high in the unstimulated neonatal cells as compared to those of the adults (log_2_ fold change ≥ 2) and showed an exacerbated response to TCR/IL-12 (Figure 6A). These genes (Figure 6B) are annotated as transcription factors, metabolism and signaling genes. MEOX1 is a transcription factor that is a member of a subfamily of homeobox genes with an important role in development and cell division. The calcium channel TRPM2 is involved in the response to TGFβ and the activation of the NLRP3 inflammasome. CD33, a transmembrane receptor with an Immunoreceptor tyrosine based inhibitory domain (ITIM) is potentially involved in negative signaling for T cell activation. CD38, a cell adhesion protein with cyclic ADP ribose hydrolase domain is involved in the regulation of intracellular calcium. These results indicate that a proportion of pro-inflammatory and innate immunity genes remained expressed in the neonatal CD8^+^ T cells, despite the transcriptional maturation induced by stimulation in the presence of IL-12. These genes account for 16.47% of the differentially expressed genes (Figure 2, clusters 1 and 6) in neonatal cells and could explain the high inflammatory response of neonates to infections.

### Changes in Promoter DNA Methylation Induced by TCR or TCR/IL-12 Signals

**Figure 7:**
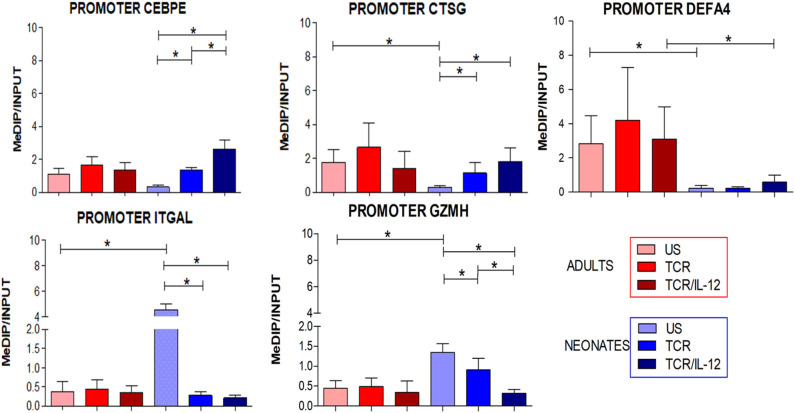
Changes in the promoter methylation induced by TCR or TCR/IL-12 signals. Histograms displaying the changes in levels of DNA methylation in the promoters of selected genes related with innate immunity vs. those of selected genes related to CD8^+^ T cell functions. Methylation was evaluated by MeDIP/qPCR of anti-Methylcytosine chromatin immuno-precipitates against non-treated DNA from the same sample (Input). Data presented are means ± standard deviation, with *n* = 6 for all assays. Statistical significance was assessed by a Student's *t*-test (unpaired; *p < 0.05).

In our previous study, we reported that the unique transcriptional signature of neonatal CD8^+^ T cells was accompanied by an epigenetic signature (10). We thus evaluated in a subset of genes that were up- or down-regulated by TCR/IL-12 whether the DNA methylation density of their promoters was altered by cell stimulation in the presence or absence of IL-12, as evaluated by MeDIP-PCR assays. Indeed, the methylation of the DNA of the promoters of the down-regulated genes *CEBPE, CTSG*, and *DEF4A* increased upon TCR stimulation, but the methylation level was significantly stronger after TCR/IL-12 treatment in the neonatal cells (Figure 7). In contrast, methylation of LFA1 integrin and Granzyme H promoter DNA diminished significantly upon stimulation, in particular by TCR/IL-12 treatment. This agrees with a transcriptional reprogramming of the neonatal cells by TCR/IL-12 stimulation accompanied by a modified epigenetic landscape.

## Conclusions

**Figure 8:**
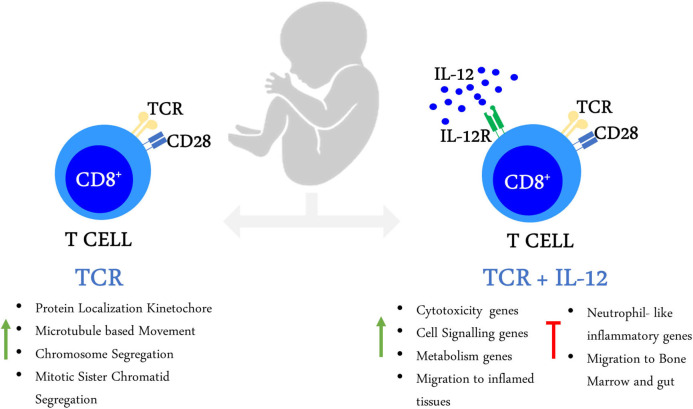
Activation of neonatal CD8^+^ T cells in the presence of IL-12 enables their transcriptional maturation.

Altogether, our results show that neonatal CD8^+^ T cells are able to reprogram their transcriptome into adult-like cells under the strong co-stimulatory conditions provided by TCR/IL-12 signals. This was achieved by reducing the expression of immaturity and neutrophil-like inflammatory genes and the over-expression of cytotoxicity and cell signaling genes; and the reprogramming of gene expression associated with metabolic and cell migration patterns. The summary of our results is shown in Figure 8.

## Discussion

In this work, we report that stimulation of neonatal cells in the presence of IL-12 induces the expression of cell signaling and cytotoxicity genes, allowing neonatal CD8^+^ T cells maturation. Furthermore, TCR/IL-12 stimulation allowed important changes in metabolism and cell functions that could explain the improved clonal expansion and function of the neonatal cells (13). We have previously shown that neonatal CD8^+^ T cells have a unique transcriptome and epigenetic signature, denoting immaturity and neutrophil-like inflammation, with low cell signaling and cytotoxicity genes activity (10). Our work shows that the presence of IL-12 during stimulation induces the transcriptional reprogramming of these unique cells, to become closer to that of adult naïve cells (Figures 1, 2). We observed that IL-12 increased the number of significantly expressed genes in both neonatal and adult cells (Figure 1). The induced genes were associated with the cytotoxic response and *bona fide* T cell functions, with the induction of IFNγ, T-bet, LFA1, Granzymes, IL-21, and FASL. IL-21 was also find induced in the presence of IL-12 in neonatal CD4^+^ T cells (30). Among the genes induced by TCR/IL-12 stimulation we found IL-10, although the receptors for this cytokine were found decreased with this high stimulatory conditions, presumably allowing the neonatal cells to decrease their high threshold of activation, while maintaining tolerance in other cells. It was also published that IL-10 is higher in the neonatal cells and is transiently induced by stimulation in the presence of IL-12 (31). Also, stimulation in the presence of IL-12 induced a permissive metabolic status for T cell activation, fostering glycolysis, NADH and NADPH metabolism, respiratory chain, and fatty acids synthesis, necessary for membrane growth. We have recently published a logical model accounting for the role of metabolic regulators in TCR activation, focusing in particular on neonatal cells (32). This study indicated that high glycolysis and reactive oxygen species in the mitochondria of neonatal CD8^+^ T cells could be responsible for the low activation of neonatal cells by TCR signals. This can be attributed to the reductive power resulting from glycolysis, presumably excreted from the neonatal cells in the form of H_2_O_2_ (32). Tentatively, the metabolic changes induced upon TCR/IL-12 stimulation could overcome this metabolic immaturity, leading to the proper activation of the cells. Chemokines also changed their expression upon TCR/IL-12 signals, CCR9 and CXCR4 that allow migration into intestines and bone marrow were downregulated, while CCR1, which permit migration into inflammatory tissues was induced. We and others have previously suggested that T cell migration is altered in neonates (33, 34), and CXCR4 was previously found expressed in neonatal CD8^+^ T cells and downregulated by strong stimulatory conditions (35).

The effect of IL-12 on neonatal cells was, however, not only related to the induction of genes but also the repression of 53% of the neonatal overexpressed genes. The down-regulated genes were associated with immaturity, the neutrophil-like response, and migration toward generative lymphoid organs and mucosal tissues. The expression of three transcription factors related to hematopoiesis and T cell development was found down-regulated, namely *HOXA3, MEOX1*, and *BCL11A*. HOXA3, and MEOX, are early and late transcription factors related to Thymocyte development, respectively (S.S. submitted). Interestingly, it is known that HOXA transcription factors play a key role in controlling cell identity and differentiation of hematopoietic stem cells and progenitors (27, 28). They are highly expressed in HSCs and progenitors, and their expression is silenced as T cells become fully mature (29) (and data not shown).

An immature and highly inflammatory profile remained, however, in the neonatal cells, as 9.54% of highly expressed neonatal genes were not responsive to stimulation (Figure 2, cluster 1). Furthermore, 6.21% of the genes had an exacerbated response to stimulation (Figure 2, cluster 5). This could explain the highly inflammatory response of neonates to infections.

Finally, we evaluated the DNA methylation status of the promoters of a subset of up- and down-regulated genes. In agreement with the inhibition of the expression of the neutrophil-related gene, the methylation of the promoters of the genes encoding for CEBPE, DEFA4, and CSTG increased in the neonatal cells upon stimulation, particularly in the presence of IL-12. CEBPE is the neutrophil signature transcription factor. In contrast, the methylation of the promoters of the induced genes coding for LFA1 and GZMH diminished. These changes were only observed in the neonatal cells, suggesting an epigenetic mechanism behind the transcriptional reprogramming induced by IL-12. Several non-coding RNAs were also induced or repressed by IL-12, several of them associated with RNA maturation and translation (Supplementary Table 2). The *NeST* lncRNA was found highly expressed cells in basal conditions but was down-regulated by the addition of IL-12 to the stimulatory conditions, only in neonatal cells. It was reported that this lncRNA epigenetically controls IFNγ gene expression (23).

STAT4, the transcription factor activated by IL-12 has been reported to control the epigenetic landscape of CD4^+^ T cells and is associated with active and repressive chromatin in CD4^+^ T cells (36). On the one hand, it induces the expression of the Mll1 methyltransferase, which is necessary for T cell differentiation into the Th1 profile and proliferation (37). On the other hand, STAT4 represses the association of DNA Methyl Transferase 3A with DNA, which is an antagonist to the Th1 phenotype (38). STAT4 is necessary for the remodeling of the CD25 locus during activation (39) and together with T-bet were recently shown to control changes in IFNγ DNA methylation (40). It will be interesting to see whether the activation of neonatal cell in the presence of IFN α/β, also activating STAT4, induces an equivalent transcriptomic change.

Altogether, our results show that stimulation in the presence of IL-12 induces a transcriptional reprogramming of neonatal CD8^+^ T cells, leading to their maturation and functionality, presumably through epigenetic mechanisms.

## Data Availability Statement

The datasets generated for this study can be found in the GSE144108 (https://www.ncbi.nlm.nih.gov/geo/query/acc.cgi?acc=GSE144108).

To review GEO accession GSE144108: Go to https://www.ncbi.nlm.nih.gov/geo/query/acc.cgi?acc=GSE144108.

## Ethics Statement

The studies involving human participants were reviewed and approved by Servicios de Salud Morelos (CONBIOÉTICA-17-CEI-001-20160329). Written informed consent to participate in this study was provided by the participants' legal guardian/next of kin.

## Author Contributions

DG-R, SS, and MS conceived experiments, analyzed the data, and wrote the manuscript. SS and MS conceived and supervised the project, revised the manuscript, and secured funding. DG-R, AC-B, LK-C, JA-F, LB, and OR-P performed experiments. DP analyzed the raw data. AM-R, MT-C, and DT provided expertise and feedback.

## Conflict of Interest

The authors declare that the research was conducted in the absence of any commercial or financial relationships that could be construed as a potential conflict of interest.

## Supplementary Material

Supplementary Figure 1 Enriched pathways in non-stimulated neonatal CD8^+^ T cells. The graph shows the 20 most enriched significant REACTOME pathways (adjusted *p* < 0.05) in neonatal as compared to adult CD8^+^ T cells, identified by GSEA from WEB-based Gene Set Analysis Toolkit (WebGestalt). No significant pathway was obtained from adult naïve CD8^+^ T cells.

Supplementary Figure 2 Heatmap of the IL-10 signaling associated genes based on KEGG pathway, for unstimulated (US), TCR, or TCR/Il-12 stimulated adult (left) or neonatal CD8^+^ T cells (triplicates).

Supplementary Figure 3 Expression of NEST (IFNG-AS1) and IFNγ during the stimulation of neonatal and adult naïve CD8^+^ T cells. Genome browser screenshots of NEST and IFNγ of the RNA-seq data corresponding to neonatal and adult cells non-stimulated or stimulated with TCR or TCR/IL-12 treatments.

Supplementary Figure 4 Genome browser screenshots and validation of genes. Genome browser screenshots of a sample of significantly expressed genes after TCR or TCR/IL-12 treatment (**A**, left), and RT-PCR evaluations (**B**, right) of the same genes in independent samples (*n* = 5), normalized to β2-microglobulin. Data presented are means ± standard deviations. Statistical significance was assessed by a Student's *t*-test (unpaired; ^*^*p* < 0.05).

Supplementary Figure 5 Genes that responded to TCR signals in neonatal CD8^+^ T cells. **(A)** heatmap and **(B)** Venn Diagrams showing the neonatal genes analyzed, that is genes that responded to TCR signals (adjusted *p* < 0.05 and log_2_ fold change ≥ 2). **(C)** Enriched GO terms returned by the DAVID software for the upregulated genes. Top 20 significant GO terms are shown. **(D)** The expression of selected genes was evaluated by RT-qPCR, normalized to the β2-microglobulin gene, in independent samples (*n* = 5). Data presented are means ± standard deviation. Statistical significance was assessed by a Student's *t*-test (unpaired; ^*^*p* < 0.05).

Supplementary Figure 6 Genes significantly downregulated by TCR/IL-12 signals in neonatal and adult CD8^+^ T cells. **(A,C)** Venn Diagrams showing overexpressed genes in the neonatal **(A)** and adult **(C)** cells, but down-regulated by TCR/IL-12. **(B,D)** heatmaps of genes significantly downregulated by TCR/IL-12 in neonatal **(B,D)** adult CD8^+^ T cells (adjusted *p* < 0.05 and log_2_ fold change ≥ 1), bars on the right display manual annotations of functional categories.

Supplementary Figure 7 Genes overexpressed in neonatal CD8^+^ T cells, which were refractory to stimulation. Heatmap with manual annotation of genes refractory to stimulation, taken from *k*-means Cluster 1, from Figure 2.

Supplementary Table 1 Lnc RNAs and Potential Functions. List of lncRNAs found in the neonatal CD8^+^ T cells and functions suggested by potential gene interactions.

Supplementary Table 2 Oligonucleotides used for PCR studies.
