# Supplementary figures and images for: IL-12 Signaling Contributes to the Reprogramming of Neonatal CD8+ T Cells
Source: Front Immunol. 2020 Jun 5;11:1089. doi: 10.3389/fimmu.2020.01089 (PMC7292210; doi:10.3389/fimmu.2020.01089)

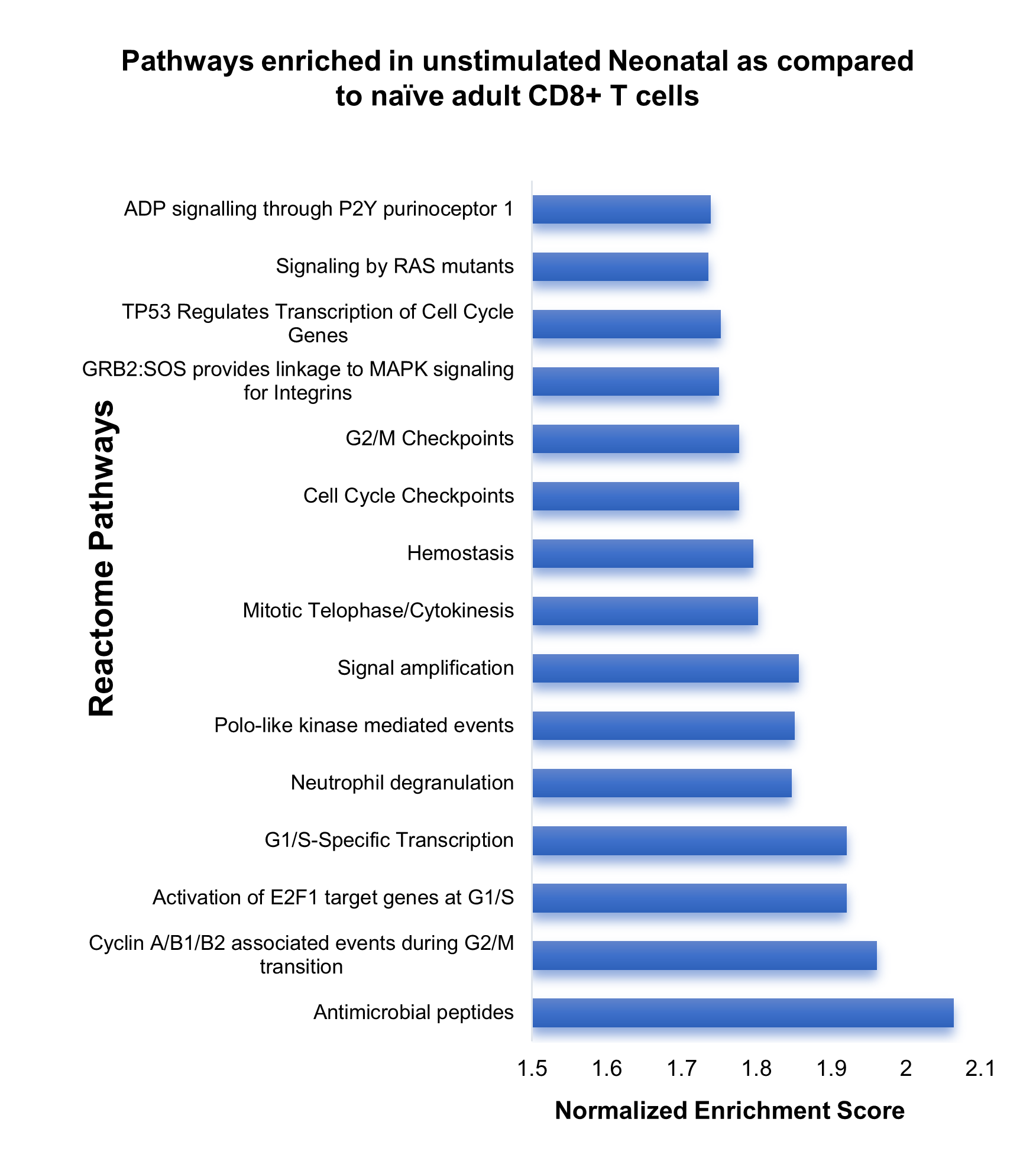

Supplement: Supplementary Figure 1 — Enriched pathways in non-stimulated neonatal CD8+ T cells. The graph shows the 20 most enriched significant REACTOME pathways (adjusted p < 0.05) in neonatal as compared to adult CD8+ T cells, identified by GSEA from WEB-based Gene Set Analysis Toolkit (WebGestalt). No significant pathway was obtained from adult naïve CD8+ T cells. [file Image_1.TIF]

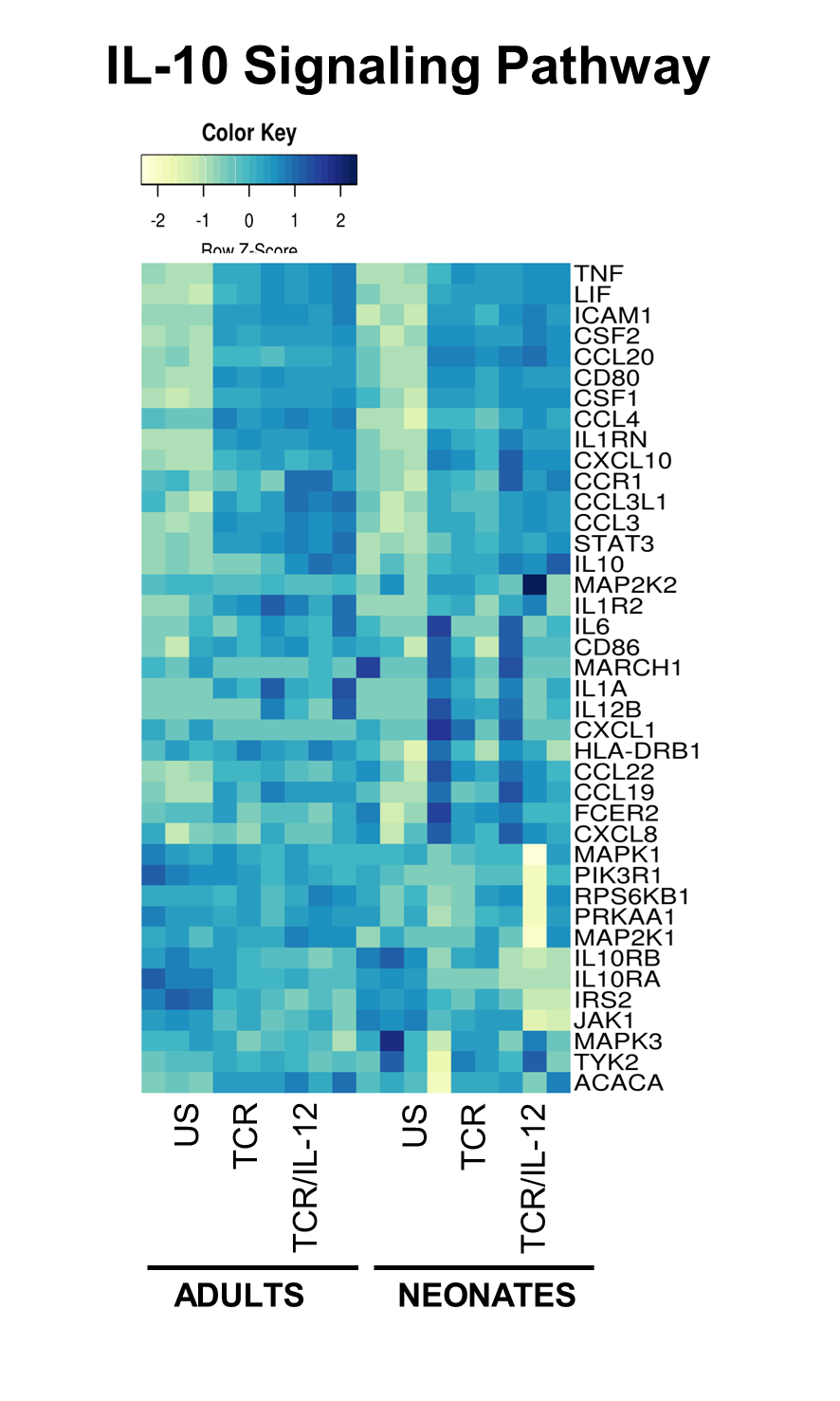

Supplement: Supplementary Figure 2 — Heatmap of the IL-10 signaling associated genes based on KEGG pathway, for unstimulated (US), TCR, or TCR/Il-12 stimulated adult (left) or neonatal CD8+ T cells (triplicates). [file Image_2.TIF]

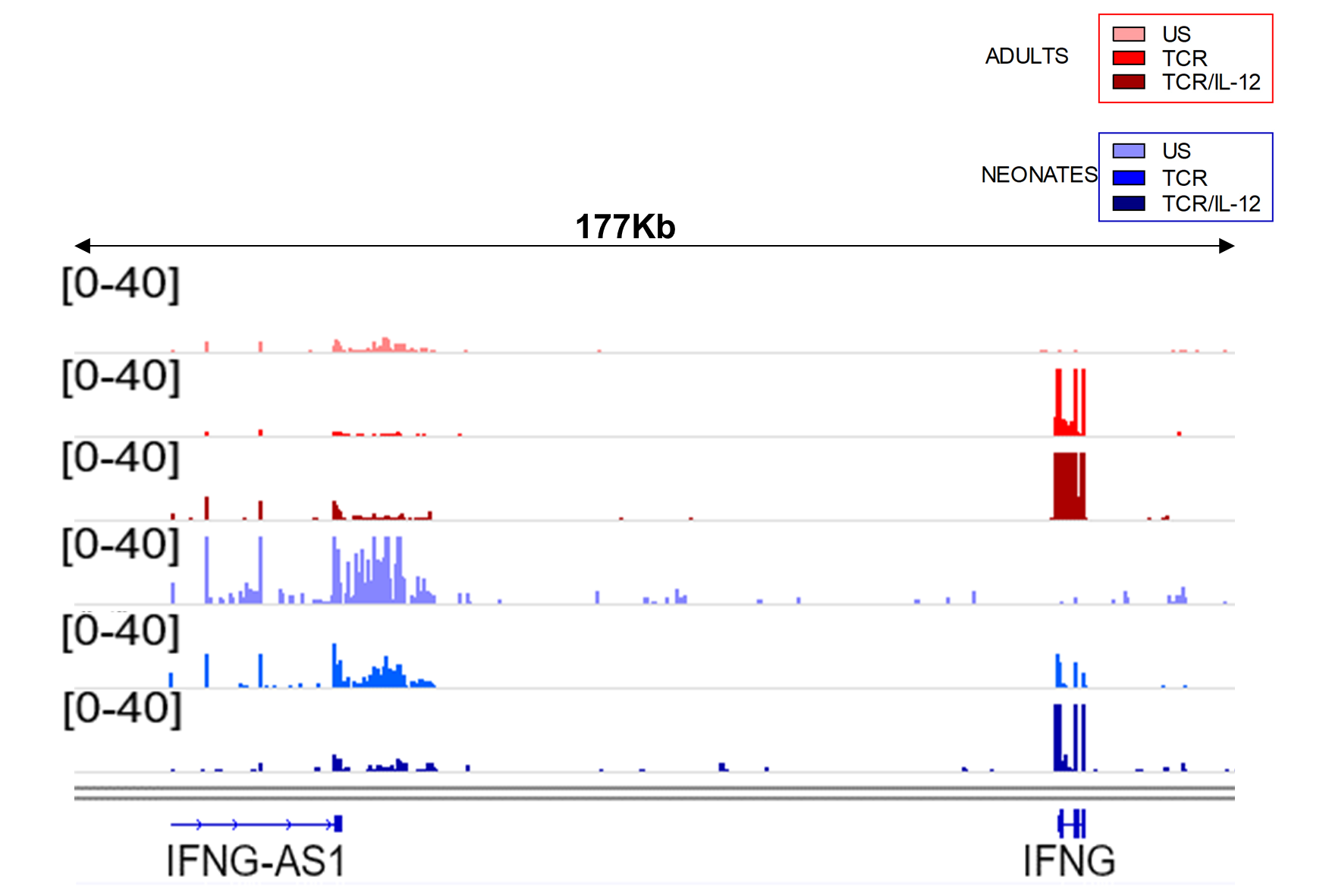

Supplement: Supplementary Figure 3 — Expression of NEST (IFNG-AS1) and IFNγ during the stimulation of neonatal and adult naïve CD8+ T cells. Genome browser screenshots of NEST and IFNγ of the RNA-seq data corresponding to neonatal and adult cells non-stimulated or stimulated with TCR or TCR/IL-12 treatments. [file Image_3.TIF]

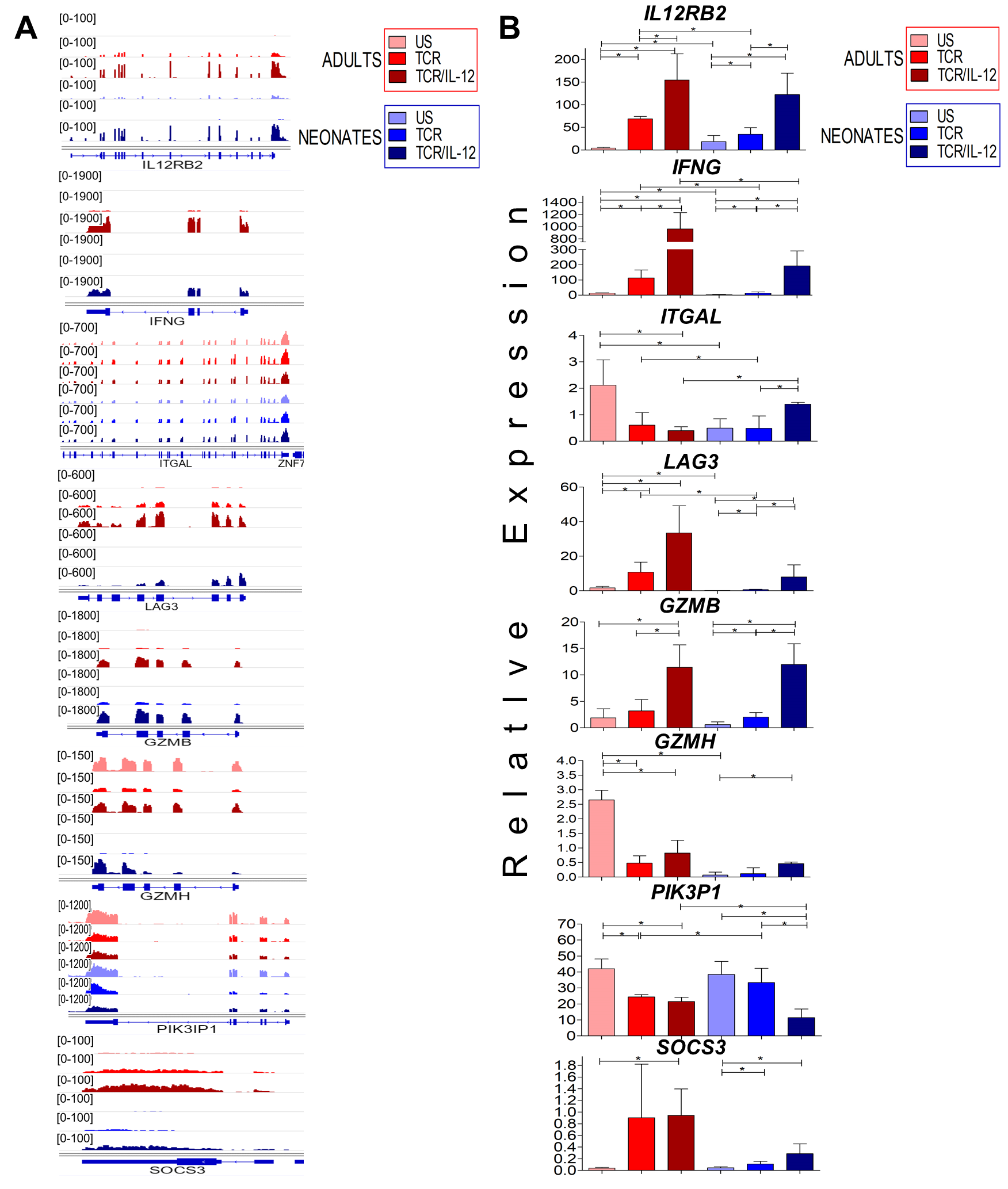

Supplement: Supplementary Figure 4 — Genome browser screenshots and validation of genes. Genome browser screenshots of a sample of significantly expressed genes after TCR or TCR/IL-12 treatment (A, left), and RT-PCR evaluations (B, right) of the same genes in independent samples (n = 5), normalized to β2-microglobulin. Data presented are means ± standard deviations. Statistical significance was assessed by a Student's t-test (unpaired; *p < 0.05). [file Image_4.TIF]

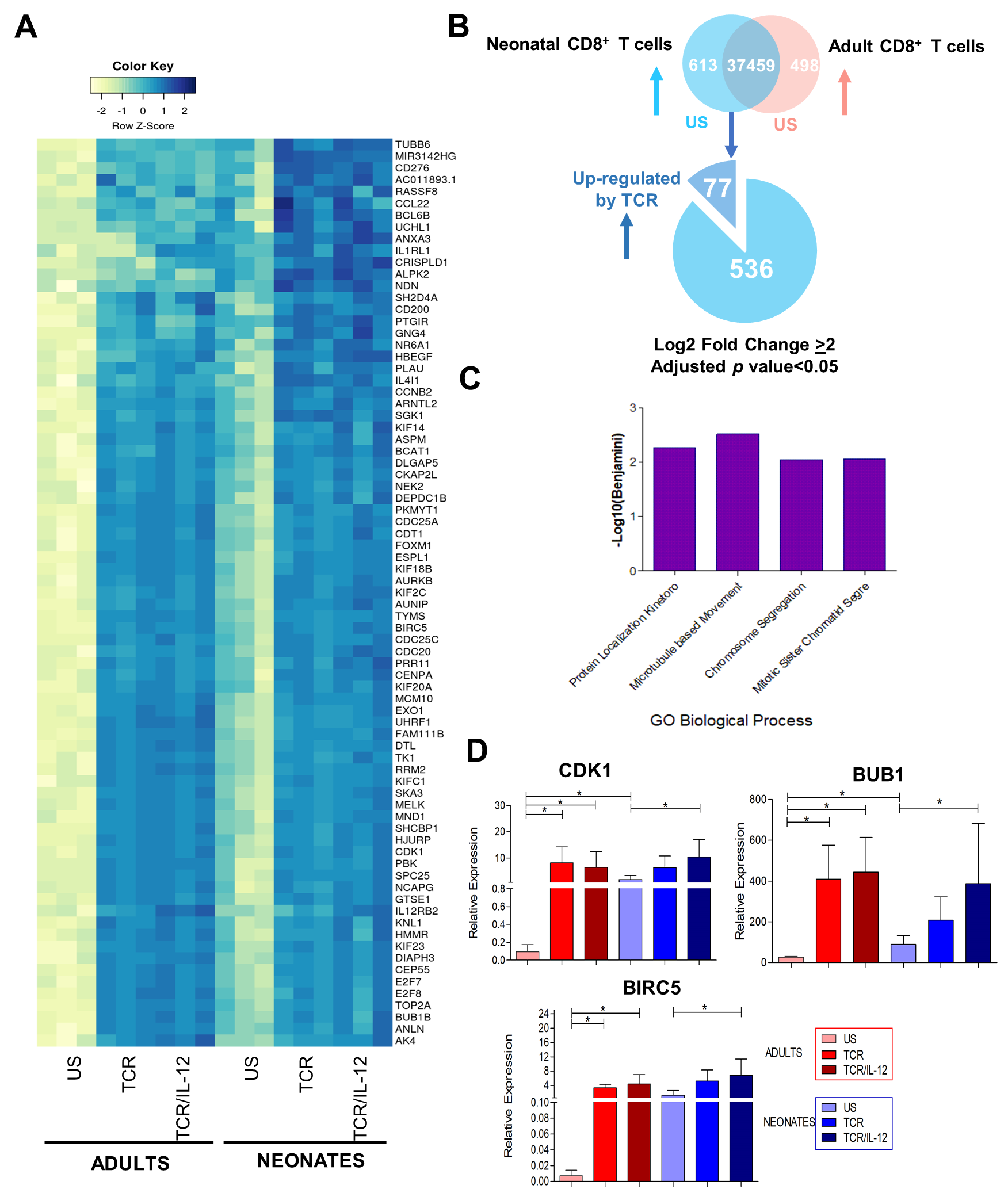

Supplement: Supplementary Figure 5 — Genes that responded to TCR signals in neonatal CD8+ T cells. (A) heatmap and (B) Venn Diagrams showing the neonatal genes analyzed, that is genes that responded to TCR signals (adjusted p < 0.05 and log2 fold change ≥ 2). (C) Enriched GO terms returned by the DAVID software for the upregulated genes. Top 20 significant GO terms are shown. (D) The expression of selected genes was evaluated by RT-qPCR, normalized to the β2-microglobulin gene, in independent samples (n = 5). Data presented are means ± standard deviation. Statistical significance was assessed by a Student's t-test (unpaired; *p < 0.05). [file Image_5.TIF]

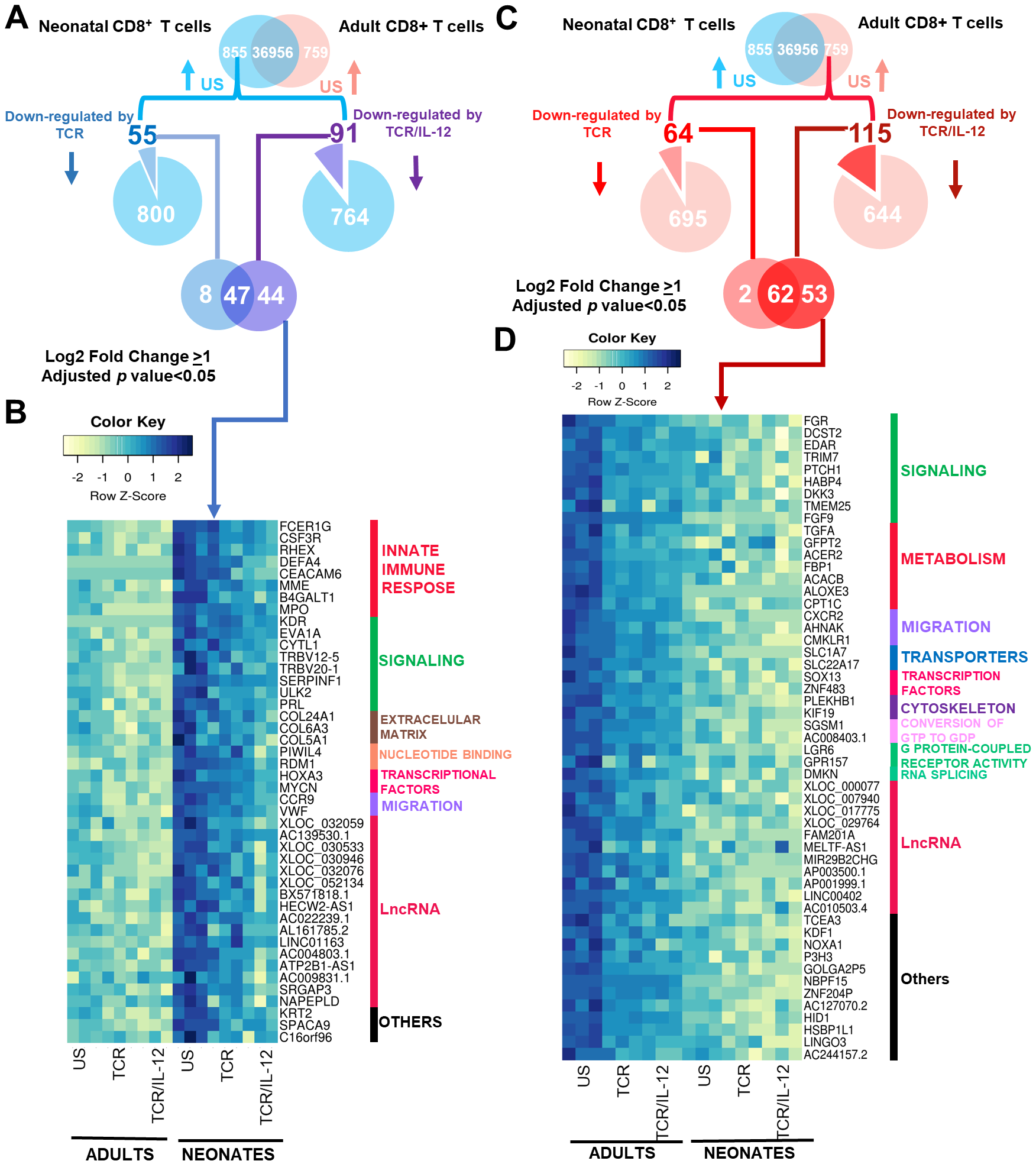

Supplement: Supplementary Figure 6 — Genes significantly downregulated by TCR/IL-12 signals in neonatal and adult CD8+ T cells. (A,C) Venn Diagrams showing overexpressed genes in the neonatal (A) and adult (C) cells, but down-regulated by TCR/IL-12. (B,D) heatmaps of genes significantly downregulated by TCR/IL-12 in neonatal (B,D) adult CD8+ T cells (adjusted p < 0.05 and log2 fold change ≥ 1), bars on the right display manual annotations of functional categories. [file Image_6.TIF]

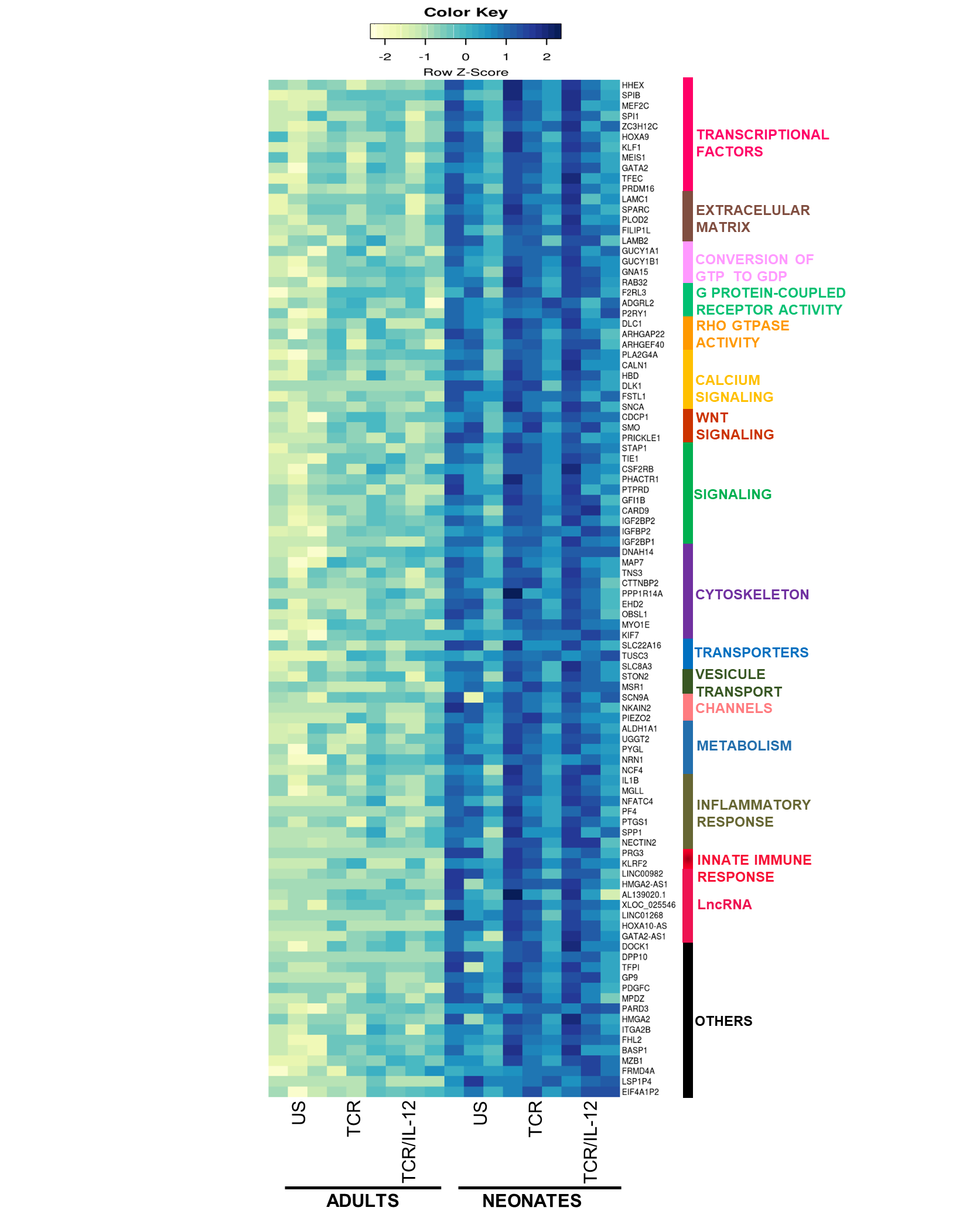

Supplement: Supplementary Figure 7 — Genes overexpressed in neonatal CD8+ T cells, which were refractory to stimulation. Heatmap with manual annotation of genes refractory to stimulation, taken from k-means Cluster 1, from Figure 2. [file Image_7.TIF]
