# Supplementary material for: IL-12 Signaling Contributes to the Reprogramming of Neonatal CD8+ T Cells
Source: Front Immunol. 2020 Jun 5;11:1089. doi: 10.3389/fimmu.2020.01089 (PMC7292210; doi:10.3389/fimmu.2020.01089)
Supplement: Supplementary Table 1 — Lnc RNAs and Potential Functions. List of lncRNAs found in the neonatal CD8+ T cells and functions suggested by potential gene interactions. [file Data_Sheet_1.PDF]

lncRNAs and Potential Functions

Gene Symbol	ENSEMBLE ID	Description	Class	Gene Interaction	Protein Interaction
XLOC_049218	NA	novel transcript	LncRNA	NA	NA
XLOC_052360	NA	novel transcript	LncRNA	NA	NA
SPATA3-AS1	ENST00000414876	SPATA3 antisense RNA 1 (head to head)	LncRNA	PPARGC1B	Peroxisome proliferator-activated receptor gamma coactivator 1-beta
TNKS2-AS1	ENST00000432246	TNKS2 antisense RNA 1 (head to head)	LncRNA	GRWD1	Glutamate-rich WD repeat-containing protein 1
LINC01160	ENST00000450155	Long intergenic non-protein coding RNA 1160	LncRNA	AQR	RNA helicase aquarius
LINC00664	ENST00000599078	long intergenic non-protein coding RNA 664	LncRNA	UBTF	Nucleolar transcription factor 1
LINC02605	ENST00000565297	long intergenic non-protein coding RNA 2605	LncRNA	PELP1	Proline-, glutamic acid- and leucine-rich protein 1
RNF144A-AS1	ENST00000437589	RNF144A antisense RNA 1	LncRNA	TDRD9	ATP-dependent RNA helicase TDRD9
CRNDE	ENST00000501177	Long Intergenic Non-Protein Coding RNA 180.	LncRNA	C1QBP	Complement component 1 Q subcomponent-binding protein, mitochondrial
AC022239.1	ENST00000527922	novel transcript, antisense to BLK	LncRNA	THOC2	THO complex subunit 2
AL161785.2	ENST00000427080	novel transcript	LncRNA	NISCH	Nischarin
BX571818.1	ENST00000585172.2	novel pseudogene	LncRNA	ABCC9	ATP-binding cassette sub-family C member 9
ERVH48-1	ENST00000447535	endogenous retrovirus group 48 member 1	LncRNA	MYT1	Myelin transcription factor 1
IL21-AS1	ENST00000417927	IL21 antisense RNA 1	LncRNA	AEBP2	Zinc finger protein AEBP2
DHCR24-DT	ENST00000443284.1	DHCR24 divergent transcript	LncRNA	CCDC180	Coiled-coil domain-containing protein 180
XLOC_032059	NA	novel transcript	LncRNA	NA	NA
XLOC_006885	NA	novel transcript	LncRNA	NA	NA
XLOC_016705	NA	novel transcript	LncRNA	NA	NA
XLOC_030946	NA	novel transcript	LncRNA	NA	NA
XLOC_032076	NA	novel transcript	LncRNA	NA	NA
