# Supplementary material for: IL-12 Signaling Contributes to the Reprogramming of Neonatal CD8+ T Cells
Source: Front Immunol. 2020 Jun 5;11:1089. doi: 10.3389/fimmu.2020.01089 (PMC7292210; doi:10.3389/fimmu.2020.01089)
Supplement: Supplementary Table 2 — Oligonucleotides used for PCR studies. [file Data_Sheet_2.PDF]

Oligonucleotides used for qPCR Amplifications

Gene name	MeDIP/RNA	Forward/Reverse	Sequence 5' --> 3'	Promoter Region
LAG3	RNA	Forward	ACACCCCATCCCAGAGGAGTT	N/A
		Reverse	AAATCGTCTTGGTCGCCACTGT	N/A
IL12RB2	RNA	Forward	CCAGCCTCAGCTCTGTGAAAT	N/A
		Reverse	GCGTTGAGAAAATGCCCACC	N/A
SOCS3	RNA	Forward	TTTCTGATCCGCGACAGCTC	N/A
		Reverse	CGGAGGAGGGTTCAGTAGGT	N/A
PIK3IP1	RNA	Forward	GCTACTCCTACAAGAGGGGGAA	N/A
		Reverse	CCTCCTGAGGGTCAACTGGA	N/A
BUB1	RNA	Forward	TCCTTCAGATGCTTGAAGCCCA	N/A
		Reverse	ACAGAGGGGATGACAGGGTTC	N/A
CDK1	RNA	Forward	TAAGCCGGGATCTACCATACCC	N/A
		Reverse	CCTGGAATCCTGCATAAGCACA	N/A
BIRC5	RNA	Forward	AGGACCACCGCATCTCTACA	N/A
		Reverse	TGTTCCTCTATGGGGTCGTCA	N/A
B2M	RNA	Forward	AGCCCAAGATAGTTAAGTGGGATCG	N/A
		Reverse	TCCAAATGCGGCATCTTCAAACC	N/A
ITGAL	RNA	Forward	CCTGTGTTACCTCTTCCGCC	N/A
		Reverse	GAACAGCAGCAAACTGGTACG	N/A
GZMH	RNA	Forward	CAGGGAAGCTCCATAAATGTCACC	N/A
		Reverse	CAGTGTGGTTGCTAAAGTGCTC	N/A
CEBPE	RNA	Forward	CACATACCTTCGGCCCAGAC	N/A
		Reverse	AAAGGGGCCTTGAGAACGC	N/A
DEFA4	RNA	Forward	ACTGCCTCATTGGTGGTGTG	N/A
		Reverse	GGCGTTCCCAGCATGACATT	N/A
IFNG	RNA	Forward	TGAATGTCCAACGCAAAGCA	N/A
		Reverse	CTGTTTTAGCTGCTGGCGAC	N/A
CTSG	RNA	Forward	GATGTGGAGGGTTCCTGGTG	N/A
		Reverse	GTGTTGCTGGGTGTTTTCCC	N/A
GZMB	RNA	Forward	AGCCTGCACCAAAGTCTTCAA	N/A
		Reverse	TTTCATTACAGCGGGGGCTT	N/A
GZMH	MeDIP	Forward	ACATGGCTCTTGCCCTTGAA	From -317 to -430
		Reverse	GTCTCACACCAACAGGCAGA	From -317 to -430
ITGAL	MeDIP	Forward	GCAAGCAGGCACAGGAAATG	From -77 to +51
		Reverse	TTCTCACAGAGGCAACAGGC	From -77 to +51
CEBPE	MeDIP	Forward	GGAACAAGCTCTACACGCGA	From -35 to -182
		Reverse	TGGAGCCACCGGAGGATTAT	From -35 to -182
CTSG	MeDIP	Forward	ATGTTCCAGACGGCTCCTTG	From -2474 to -2604
		Reverse	CCCATTGTTGAGTATGCGGC	From -2474 to -2604
DEFA4	MeDIP	Forward	AAATGCCACCCACACACAGA	From -407 to -577
		Reverse	CAACCTGCCCTATCCAGCAA	From -407 to -577
